# Malaria is a cause of iron deficiency in African children

**DOI:** 10.1038/s41591-021-01238-4

**Published:** 2021-02-22

**Authors:** John Muthii Muriuki, Alexander J. Mentzer, Ruth Mitchell, Emily L. Webb, Anthony O. Etyang, Catherine Kyobutungi, Alireza Morovat, Wandia Kimita, Francis M. Ndungu, Alex W. Macharia, Caroline J. Ngetsa, Johnstone Makale, Swaib A. Lule, Solomon K. Musani, Laura M. Raffield, Clare L. Cutland, Sodiomon B. Sirima, Amidou Diarra, Alfred B. Tiono, Michal Fried, Moses Gwamaka, Adu-Afarwuah Seth, James P. Wirth, Rita Wegmüller, Shabir A. Madhi, Robert W. Snow, Adrian V.S. Hill, Kirk A. Rockett, Manjinder S. Sandhu, Dominic P. Kwiatkowski, Andrew M. Prentice, Kendra A. Byrd, Alex Ndjebayi, Christine P. Stewart, Reina Engle-Stone, Tim J. Green, Crystal D. Karakochuk, Parminder S. Suchdev, Philip Bejon, Patrick E. Duffy, George Davey Smith, Alison M. Elliott, Thomas N. Williams, Sarah H. Atkinson

**Affiliations:** 1 Kenya Medical Research Institute (KEMRI), Centre for Geographic Medicine Research, Coast, KEMRI-Wellcome Trust Research Programme, Kilifi, Kenya; 2 Open University, KEMRI-Wellcome Trust Research Programme, Accredited Research Centre, Kilifi, Kenya; 3 Wellcome Centre for Human Genetics, Nuffield Department of Medicine, University of Oxford, Oxford, UK; 4 Big Data Institute, Li Ka Shing Centre for Health Information and Discovery, University of Oxford, Oxford, UK; 5 Medical Research Council (MRC) Integrative Epidemiology Unit, Population Health Sciences, Bristol Medical School, University of Bristol, Bristol, UK; 6 MRC Tropical Epidemiology Group, Department of Infectious Disease Epidemiology, London School of Hygiene and Tropical Medicine, London, UK; 7 African Population and Health Research Centre, Nairobi, Kenya; 8 Department of Clinical Biochemistry, Oxford University Hospitals, Oxford, UK; 9 MRC/Uganda Virus Research Institute and London School of Hygiene and Tropical Medicine Uganda Research Unit, Entebbe, Uganda; 10 Department of Medicine, University of Mississippi Medical Center, Jackson, MS, USA; 11 Department of Genetics, University of North Carolina, Chapel Hill, NC, USA; 12 South African Medical Research Council: Vaccines and Infectious Diseases Analytical Research Unit, Faculty of Health Sciences, University of the Witwatersrand, Johannesburg, South Africa; 13 Groupe de Recherche Action en Sante (GRAS), 06 BP 10248, Ouagadougou, Burkina Faso; 14 Laboratory of Malaria Immunology and Vaccinology, National Institute of Allergy and Infectious Diseases, National Institutes of Health, Bethesda, MD, USA; 15 Mother Offspring Malaria Studies (MOMS) Project, Seattle Biomedical Research Institute, Seattle, WA, USA; 16 Muheza Designated District Hospital, Muheza, Tanzania; 17 University of Dar es Salaam, Mbeya College of Health and Allied Sciences, Mbeya, Tanzania; 18 Department of Nutrition and Food Science, University of Ghana, Legon, Ghana; 19 GroundWork, Fläsch, Switzerland; 20 Centre for Tropical Medicine and Global Health, Nuffield Department of Medicine University of Oxford, Oxford, UK; 21 ’Centre for Clinical Vaccinology and Tropical Medicine and the Jenner Institute Laboratories, University of Oxford, Oxford, UK; 22 Wellcome Sanger Institute, Hinxton, UK; 23 MRC Unit The Gambia at London School of Hygiene and Tropical Medicine, Banjul, The Gambia; 24 WorldFish, Bayan Lepas, Malaysia; 25 Helen Keller International, Yaoundé, Cameroon; 26 Department of Nutrition, University of California, Davis, Davis, CA, USA; 27 SAHMRi Women and Kids, South Australian Health and Medical Research Institute, Adelaide, South Australia, Australia; 28 School of Medicine, The University of Adelaide, Adelaide, South Australia, Australia; 29 Food, Nutrition, and Health, University of British Columbia, Vancouver, British Columbia, Canada; 30 Department of Pediatrics, Emory University and Emory Global Health Institute, Atlanta, GA, USA; 31 Department of Clinical Research, London School of Hygiene and Tropical Medicine, London, UK; 32 Department of Infectious Diseases and Institute of Global Health Innovation, Imperial College, London, UK; 33 Department of Paediatrics, University of Oxford, Oxford, UK

## Abstract

Malaria and iron deficiency (ID) are common and interrelated public health problems in African children. Observational data suggest that interrupting malaria transmission reduces the prevalence of ID^1^. To test the hypothesis that malaria might cause ID, we used sickle cell trait (HbAS, rs334), a genetic variant that confers specific protection against malaria^2^, as an instrumental variable in Mendelian randomization analyses. HbAS was associated with a 30% reduction in ID among children living in malaria-endemic countries in Africa (*n* =7,453), but not among individuals living in malaria-free areas (*n =3,818*). Genetically predicted malaria risk was associated with an odds ratio of 2.65 for ID per unit increase in the log incidence rate of malaria. This suggests that an intervention that halves the risk of malaria episodes would reduce the prevalence of ID in African children by 49%.

Malaria and ID are important co-existing public health problems across sub-Saharan Africa, and their relationship is complex and incompletely understood. Malaria transmission is persistent and widespread across sub-Saharan Africa with an estimated parasite prevalence of 24% (ref. ^3^) and 213 million cases in 2018 (ref. ^4^). Similarly, ID is also common and accounts for over 60% of anemia cases ^5^. ID is associated with impaired cognitive development ^6^ and is a leading cause of years lived with disability in African children^7^. ID may be caused by a number of factors, including diets low in available iron, dietary iron inhibitors, such as polyphenols and phytates, and helminth infections. Iron supplementation, via tablets or syrups and micronutrient powders, are the primary interventions to manage ID and anemia in children. However, there are long-standing concerns regarding the safety and efficacy of iron supplements in malaria-endemic countries, where they may predispose individuals to malaria and other infections^8^ and be poorly absorbed^9,10^. New strategies are needed for the management of ID.

Malaria is known to cause anemia via various mechanisms, including the destruction of parasitized and non-parasitized erythrocytes. This process affects hemoglobin concentrations, but the iron released from destroyed erythrocytes is not lost from the body and can be recycled. Little is known about whether malaria might be a cause of ID in African children^11^. A study in the Kenyan highlands showed that interrupting malaria transmission reduced the prevalence of ID^1^, and studies in Gambian and Kenyan children showed that the prevalence of ID increased over the malaria season^12^. Moreover, stable isotope studies show that treatment of afebrile malaria increased dietary iron absorption and reduced the prevalence of ID in 17 Ivorian children and 23 Beninese women^9,10^. These studies suggest that malaria control could be an effective strategy for managing ID in sub-Saharan Africa. However, observational studies are susceptible to reverse causation and confounding, making causal inferences harder to obtain. Here, we used a Mendelian randomization approach^13^ that reduces these biases and offers a method to test for causality by studying the prevalence of ID in children whose risk of malaria is reduced by a known genetic variant (termed an instrumental variable) compared to children lacking such protection.

We used sickle cell trait (HbAS, rs334), a genetic variant that confers specific protection against all forms of clinical *Plasmodium falciparu* *m* malaria^2^, as an instrumental variable in Mendelian randomization analyses. To do this, we used data and stored samples from 7,453 healthy children aged 0–8 years (median age, 2.08 years; interquartile range, 1.67–3.25 years) from ten community-based cohorts in malaria-endemic areas in sub-Saharan Africa (Fig. 1a). We also tested whether HbAS might influence risk of ID via a mechanism that is independent of its effect on malaria (horizontal pleiotropy) in malaria-free populations, including 3,207 African Americans (21–93 years old) and 611 life-long Nairobi residents (10–16 years old) using no-relevance point sensitivity analyses^14^. We were unable to test for pleiotropy among children living in South Africa, another malaria-free area, because, like most populations without historical exposure to malaria, few had HbAS (*n=11 of 845*). These studies are described in detail in the Methods. We also considered using other polymorphisms, including glucose-6-phosphate dehydrogenase (G6PD) A deficiency and α-thalassemia, as instrumental variables, but, in meta-analyses of published literature (Extended Data Fig. 1 and Supplementary Table 1), the protective effects of these polymorphisms are largely confined to severe malaria, an outcome of limited relevance as a cause of ID at a population level. ID was defined according to the World Health Organization (WHO)guidelines as levels of ferritin <12µgl^-1^ or <30µgl^-1^ in the presence of inflammation (defined as C-reactive protein (CRP) >5mgl^-1^ or α1-antichymotrypsin (ACT) >0.6gl^-1^ or α1-acid glycoprotein (AGP) >1gl^-1^) in children <5 years old or <15 μgl^-1^ in children <5 years old^15^and as ferritin levels <30µgl^-1^ in African American adults and Nairobi school children^16^. Anemia was defined as hemoglobin levels <110g/l^-1^ in children aged <5 years, <115gl^-1^ in children ≥5 years, <120gl^-1^ in women and <130gdl^-1^ in men and ID anemia (IDA) as the presence of ID and anemia^17^. The characteristics of the study populations are summarized inTable 1.

We first evaluated the effect of HbAS on the risk of ID. Prevalence of malaria parasitemia, HbAS and ID varied across study sites (Fig. 1b). Overall prevalence of ID and HbAS was 26.93% and 13.42%, respectively. In a meta-analysis of ten community-based studies of children living in malaria-endemic areas, HbAS was associated with 30% protection from ID (odds ratio (OR), 0.70; 0.58, 0.82; Fig. 1c) and 31% protection against IDA (OR, 0.69; 0.53, 0.85; Extended Data Fig. 2), and this protective effect on ID was consistent across cohorts (*I* ^2^ = 0.0%, Fig. 1c). We found no evidence of an association between HbAS and potential confounders, such as age, sex or underweight (Fig. 1d). As HbAS and α-thalassemia show negative epistasis in their malaria-protective effects^18^, we further adjusted for the effect of α-thalassemia, but this did not alter the effect of HbAS on risk of ID (Supplementary Table 2). HbAS was similarly associated with protection against ID in pooled analyses (OR, 0.79; 0.68, 0.93) and after ferritin levels were adjusted for inflammation (OR, 0.77; 0.65, 0.89; Extended Data Fig. 3) using a regression-correction approach^19^. Prevalences of ID and IDA were similarly lower in African children carrying HbAS compared to those carrying wild-type normal hemoglobin (HbAA) (Fig. 2a,b). In populations not exposed to malaria (that is, African Americans and life-long Nairobi residents), HbAS was not associated with ID (OR, 0.93; 0.55, 1.31; *P* = 0.63; Figs. 1c and 2a) or IDA (OR, 1.17; 0.56, 1.78; *P* = 0.84; Fig. 2band Extended Data Fig. 2), suggesting that HbAS was protective against ID only in malaria-endemic populations.

We then estimated the causal effect of malaria on ID using HbAS as an instrumental variable for malaria in a two-sample Mendelian randomization analysis^13^. First, to determine the effect of HbAS on uncomplicated malaria, we conducted a systematic review and meta-analysis of all published papers that assessed the effect of HbAS on the incidence of uncomplicated malaria in African populations to obtain an overall incidence rate ratio (IRR) (Extended Data Fig. 4and Supplementary Table 1). Overall, HbAS was associated with 31% protection from episodes of uncomplicated malaria (IRR = 0.69; 0.64, 0.74, Extended Data Fig. 4), and the *F* statistic was 85.56. We estimated the causal log odds as the ratio of the log odds of HbAS on ID (Fig. 1c) to log incidence rate of HbAS on uncomplicated malaria (Extended Data Fig. 4) with a Mendelian randomization approach using the Wald ratio^13^. Estimates and standard errors are shown in Table 2. The causal OR was calculated by exponentiating the causal log OR (0.97). We observed a causal OR of 2.65 (95% confidence interval (CI), 1.64, 4.26; *P* = 0.0001), suggesting that the genetically predicted risk of uncomplicated malaria is associated with 2.65-fold higher odds of ID per unit increase in the log incidence rate of malaria. We then calculated the effect on ID of reducing malaria incidence by half. We obtained this by multiplying the causal log odds (0.97) by the natural logarithm of 0.5 and then exponentiated the result^20^. We observed an OR of 0.51, suggesting that reducing the incidence of uncomplicated malaria by half would reduce ID by 49%. In sum, our findings suggest that malaria is an important cause of ID and that malaria control may be an effective strategy for addressing ID among children living in sub-Saharan Africa.

So how might malaria cause ID? A number of pathways might be involved, including urinary iron loss from hemolysis and the induction of inflammation. We found that children with afebrile and severe malaria had higher geometric mean CRP levels (4.62mgl^-1^ and 100.28mgl^-1^,respectively) compared to levels in those without malaria (1.15mgl^-1^). Carriage of HbAS was associated with a 25% reduction in inflammation (OR, 0.75; 0.65, 0.87; *P=0.0002*;Figs. 1dand 2d) in African children but not in malaria-free populations (OR, 0.98; 0.75, 1.28; *P=0.87*;Fig. 2d). The iron hormone, hepcidin, is upregulated by inflammation and downregulated by ID and increased erythropoietic drive in African children^12^. Hepcidin causes ID by blocking duodenal iron absorption and macrophage iron recycling by degrading the iron transporter ferro-portin^21^. Malaria is known to strongly upregulate the production of hepcidin by the liver^22,23^, and we similarly found that hepcidin concentrations were markedly increased in afebrile and severe malaria, above a threshold associated with reduced iron absorption^24,25^ (Fig. 2c). How this might lead to ID is illustrated in Fig. 2. Malaria might also increase hepcidin concentrations via non-inflammatory pathways (Extended Data Fig. 5). We found that children with malaria parasitemia had higher concentrations of hepcidin, above a threshold blocking iron absorption, at almost every decile of CRP compared to levels in children without malaria (Fig. 2e), and this effect was observed regardless of the presence of inflammation (Extended Data Fig. 6). Thus, HbAS carriers would avoid malaria-induced hepcidin-mediated blockade of iron absorption, leading to improved iron status. In sum, our findings suggest that malaria may drive a hepcidin-mediated block in iron absorption leading to ID, in agreement with previous studies^9,10,26^.

Our study had a number of strengths and limitations. A strength of the study was that we used large-scale datasets from populations across sub-Saharan Africa. We also used an instrumental variable, HbAS, known to confer specific and strong protection from malaria, and could therefore proxy health benefits of malaria control^27^. One limitation of our study was the use of African American adults and Nairobi school children, who might have different and age-related causes of ID, to test whether HbAS might influence ID through a non-malaria-related pathway. Further limitations were that we did not have data on other potential causes of ID in the various populations, such as differences in diet, risk of hookworm infection or other infections, such as human immunodeficiency virus. Studies also differed in the assays used to determine ferritin levels and inflammation and in their study design; for example, HbAS might be less likely to protect from ID in longitudinal studies in which children are regularly monitored and treated for malaria. Nevertheless, despite these differences, HbAS remained consistently associated with protection from ID across all of the studies in malaria-endemic areas. Another potential limitation is the uncertainty in estimating ID in the context of a high burden of infectious disease, and we therefore also defined ID using ferritin levels that were regression corrected for inflammation^19^ and found similar results. HbAS might have been expected to have a larger protective effect against ID in populations with higher exposure to malaria^28^; however, we were unable to test for this, as we did not have data on the incidence of clinical malaria in most of the studies. It is also likely that our analyses may have underestimated the effect of malaria on ID, as HbAS is not known to protect against afebrile malaria parasitemia^2^. As afebrile parasitemia increases hepcidin concentrations (Fig. 2c), is highly prevalent^3^ and is less likely to be treated, it may significantly impair iron absorption^9,10^.

In summary, using large-scale data (*n=11,333*) from children across the African continent and from African Americans, we provide evidence that malaria increases the risk of ID in African children (OR = 2.65; 1.64, 4.26). The public health benefits of malaria control in terms of reducing ID depend on the efficacy of the specific interventions used. Our data suggest that an intervention that halves the incidence of malaria would reduce ID by 49%. In a small study of Ivorian children, the prevalence of ID was reduced by half following treatment for afebrile malaria^9^, suggesting that the predicted benefits from our analysis may be translatable to the real world. Studies in malaria-endemic areas have focused on the potential for iron status to influence the risk of malaria infection. These studies have led to questioning whether iron supplementation can be safely given in the management of ID in malaria-endemic areas^8,29^. However, our findings suggest that malaria itself may be causing ID in African children. Other infections may similarly cause ID; for example, respiratory infections were associated with hepcidin-mediated ID in Gambian children^30^. The management of ID has traditionally involved iron supplementation. However, in addition to long-standing concerns regarding the safety of iron supplementation in Africa, there are also concerns regarding effects on the gut microbiome^31^ and lack of efficacy in areas of high infectious burden where chronically raised hepcidin levels may inhibit iron absorption^9,10^. Interventions that reduce malaria would allow iron to be more effectively absorbed from dietary sources. We recommend that strategies to prevent and treat malaria and other infections should be an integral part of programs to control ID in African children. Future research should confirm these findings by conducting trials of malaria control, for example, intermittent preventative treatment trials of malaria to evaluate the effect of malaria control on iron status.

## Methods

### Study populations and laboratory methods

This study included ten cohorts of healthy children (in Malawi, Ghana, Burkina Faso, DRC, Kenya (Kilifi and Western), Tanzania, The Gambia, Cameroon and Uganda) living in malaria-endemic countries. Four of these studies (in Malawi, Ghana, DRC and The Gambia) were cross-sectional studies, two were longitudinal (in Tanzania and Kilifi Kenya), two were RCT (in Burkina Faso and Uganda), one was a cluster RCT (in Western Kenya), and one was a cluster survey (in Cameroon). The study also included three cohorts living in countries with no malaria exposure, African American adults from the longitudinal JHS, children from Soweto, South Africa and a survey of school children in Nairobi, Kenya. These studies are described below.

### Malaria-endemic study sites

#### Malawi

The 2015–2016 Malawi Micronutrient Survey (MMS) was conducted as part of the Malawi Demographic and Health Survey. This cross-sectional survey aimed to determine the prevalence of anemia, micronutrient deficiencies (iron and vitamin A), infections and hemoglobinopathies^32^. The MMS included all children aged 6–59 months from randomly selected clusters and households. Details of the study design are available elsewhere^32^. Briefly, whole blood collected in tubes containing EDTA was used to test for malaria using a rapid diagnostic test (RDT) (SD Bioline Malaria *P. falciparum* (HRP2), Alere) and to measure hemoglobin concentrations using the HemoCue 301 system (HemoCue America). Levels of serum ferritin, CRP and AGP were measured using sandwich ELISA (VitMin Laboratory)^33^. Genotyping of sickle cell trait and α-thalassemia was performed using PCR as previously described^32^.

#### Ghana

This study was part of the 2017 Ghana Micronutrient Survey. Details of the cross-sectional study design and ethical approvals are presented in ref.^34^. Children aged 6–59 months were recruited from three strata (Southern Belt, Middle Belt and Northern Belt) in Ghana, and random selection was performed in each stratum. Blood sampling and anthropometry were conducted during the survey. Malaria testing was performed using RDT (SD Bioline Malaria Ag Pf/Pan RDT kit (Standard Diagnostics); hemoglobin concentrations were measured using HemoCue 301 AB). Sandwich ELISA^33^ was used to measure serum ferritin, CRP and AGP concentrations. DNA was extracted from blood pellets and used to type sickle cell trait and α-thalassemia using PCR^35,36^.

#### Burkina Faso

##### The VAC050 ME-TRAP malaria vaccine trial

This was part of the VaccGene study, which aimed to identify genetic variants associated with differential response to vaccination in infancy but with ethical approval to undertake analyses to examine the effect of iron status on infection susceptibility. Details of the study design are described elsewhere^37^. Infants between the ages of 6 and 18 months living in the Banfora region of Burkina Faso were recruited into a phase 1–2b clinical trial to test the safety, immunogenicity and efficacy of an experimental heterologous viral-vectored prime-boost liver-stage malaria vaccine^37^. Serum ferritin (Chemiluminescent Microparticle Immunoassay, Abbott Architect), hepcidin (DRG Hepcidin 25 (bioactive) High Sensitive ELISA kit (DRG International)), CRP (Multigent CRP Vario assay, Abbott Architect), hemoglobin (Coulter analyzer, Beckman Coulter) and malaria parasitemia (Giemsa-stained thick and thin blood films) were measured at a single time point. Genotyping of sickle cell trait in the VaccGene study is described below.

#### Western Kenya

Children were recruited from rural villages in Bungoma, Kakamega and Vihiga counties in western Kenya, using a cluster study design as part of the water, sanitation and handwashing benefits RCT (WASH Benefits trial). Details of the study design were published elsewhere^38^. Samples collected from the environmental enteropathy endline survey were used. Venous blood samples were used to test for malaria parasitemia using RDT (SD Bioline Malaria *P. falciparum* (HRP2), Alere) and hemoglobin concentrations (HemoCue Hb 301). Serum ferritin and CRP levels were assayed using sandwich ELISA^33^. Serum hepcidin-25 levels were quantified by using a competitive ELISA kit (PenLabs). Genotyping of sickle hemoglobin types and α-thalassemia was conducted using PCR^35,36^.

#### Sud Kivu and Kongo Central, Democratic Republic of Congo

This study used data collected during a nutrition cross-sectional survey of mothers and their children aged 6–59 months in rural Sud Kivu and Kongo Central provinces in the DRC as described elsewhere^39^. Venous blood samples were used to test for malaria parasitemia using RDT (CareStart Malaria Screen, Access Bio). Serum ferritin, CRP and AGP levels were assayed using sandwich ELISA^33^. Hemoglobin typing for sickle cell trait was conducted using pyrosequencing, while PCR was used to detect α-thalassemia as described elsewhere^39^.

#### Kilifi, Kenya

This was an ongoing rolling longitudinal study designed to evaluate immunity to malaria in children and is described elsewhere^40^. Within this cohort, children were followed to 8 years of age with weekly follow-ups and annual cross-sectional surveys during which anthropometry measurements were made and blood samples were collected. Serum ferritin (Chemiluminescent Microparticle Immunoassay, Abbott Architect), hepcidin (DRG Hepcidin 25 (bioactive) High Sensitive ELISA kit (DRG International)), CRP (Multigent CRP Vario assay, Abbott Architect), hemoglobin (Coulter analyzer, Beckman Coulter) and malaria parasitemia (Giemsa-stained thick and thin blood films) were measured from blood samples collected at a single cross-sectional survey based on the availability of plasma samples archived at 80°C. Genotyping of hemoglobin types and α-thalassemia was conducted by PCR^35,36^ using DNA extracted with the Qiagen DNA Blood Mini kit (Qiagen).

#### Muheza, Tanzania

Children were enrolled at delivery into the MOMS Project longitudinal birth cohort at Muheza District Hospital in northeastern Tanzania between 2002 and 2006. Children were assessed for malaria parasitemia every 2 weeks during infancy and monthly thereafter, as well as at the time of any illness. Singleton children without evidence of human immunodeficiency virus in themselves or in their mothers during follow-up were included in studies of iron status and risk of malaria infection as described elsewhere^41^. Blood samples were collected at 3, 6 and 12 months of age and then once every 6 months in years 2 and 3^41^. In this study, we used data from samples collected at a single time point, each child’s oldest time point, as older children were likely to have experienced more malaria episodes. The presence of *P. falciparum* parasitemia was determined using Giemsa-stained thick blood smears, while hemoglobin levels were measured using an impedance-based analyzer (Abbott Cell Dyn 1200). Plasma ferritin and CRP levels were assayed using a multiplex bead-based platform (Bio-Rad) and custom assay kits, and hemoglobin was typed by electrophoresis (Helena Laboratories)^41^. Genotyping for α-thalassemia was conducted as described by Chong et al.^36^.

#### The Gambia

##### West Kiang study

All children aged 2–6 years old were recruited from ten rural villages in the West Kiang region of The Gambia during the malaria season (July to August 2001)^42^. We used cross-sectional data collected at the start of the malaria season. All children had a clinical examination, anthropometric measurements and a 3-d course of mebendazole for possible hookworm infection. A blood sample was collected for complete blood count, observations with a malaria slide, measurements of ferritin (Microparticle Enzyme Immunoassay (Abbott Architect)), hepcidin (Hepcidin-25 (human) EIA Kit (Bachem) and ACT (immunoturbidimetry, Cobas Mira Plus Bio-analyzer, Roche) levels and DNA extraction. Children with a temperature >37.5°C had a malaria blood film, appropriate clinical treatment and a blood sample 2 weeks later after recovery from illness. Genotyping of sickle cell was performed on amplified DNA as detailed elsewhere^42^. The Gambian Bachem hepcidin values were harmonized by converting to the old DRG hepcidin assay values ((0.266×Bachem values)+1.633) and then to the new High Sensitive DRG hepcidin assay values ((1.989× old DRG values)-3.24) as previously validated^43^.

#### Yaoundé and Douala, Cameroon

Children 12–59 months of age were recruited to a cluster survey that aimed to determine the prevalence of inherited hemoglobin disorders in Yaoundé and Douala, Cameroon, as described elsewhere^44^. Venous blood samples were collected for malaria testing using RDT (SD Bioline Malaria Ag Pf/Pan, Standard Diagnostics) and hemoglobin measurement using a photometer (HemoCue). Plasma ferritin, CRP and AGP levels were assayed by ELISA^33^. Hemoglobin genotypes were determined by HPLC using an ultra-Resolution Variants Analyzer (Trinity Biotech), while α-thalassemia type was determined by PCR^44^.

#### Uganda

##### The Entebbe Mother and Baby Study (EMaBS)

EMaBS is a prospective birth cohort that was originally designed as an RCT to test whether anthelminthic treatment during pregnancy and early childhood was associated with differential response to vaccination or incidence of infections, such as pneumonia, diarrhea or malaria (http://emabs.lshtm.ac.uk/)^45^. This cohort was part of the VaccGene study as described elsewhere^46^. Blood samples were collected in Vacutainer tubes containing EDTA at birth and at subsequent birthdays up to 5 years of age. Anthropometry and biomarkers of iron and inflammation were measured in samples from a single annual visit based on the availability of stored samples. Serum ferritin (Chemiluminescent Microparticle Immunoassay, Abbott Architect), CRP (Multigent CRP Vario assay, Abbott Architect), hepcidin (DRG Hepcidin-25 (bioactive) High Sensitive ELISA kit (DRG International)), hemoglobin (Coulter analyzer, Beckman Coulter) and malaria parasitemia (Giemsa-stained thick and thin blood films) were measured. Genotyping of sickle cell trait in the VaccGene study is described below.

## Malaria-free study sites

### South Africa

#### The Soweto Vaccine Response Study

Infants born in Chris Hani Baragwanath Hospital living in the Soweto region of Johannesburg, South Africa were recruited from vaccine trials^47^ coordinated by the Respiratory and Meningeal Pathogens Unit (http://www.rmpru.com/). Mothers of the infants were approached if the infants had received all of their EPI vaccines up to 6 months of age. The infants were sampled prospectively at 12 months after receipt of the measles vaccine at 9 months of age. Single whole-blood samples were collected in vacutainer tubes containing EDTA for measurement of iron and inflammatory markers and DNA extraction. Serum ferritin (Chemiluminescent Microparticle Immunoassay, Abbott Architect), CRP (Multigent CRP Vario assay, Abbott Architect) and hepcidin (DRG Hepcidin-25 (bioactive) High Sensitive ELISA kit (DRG International)) levels were measured. This cohort was part of the VaccGene study, and genotyping is described below.

### Jackson Heart Study

This is a population-based longitudinal study of African Americans aged ≥21 years living in the Jackson, Mississippi metropolitan area in the USA^48^. This study was designed to evaluate risks of cardiovascular disease as described elsewhere^48,49^. Serum ferritin (Roche immunoturbidimetric assay), CRP (ELISA) and hemoglobin (Coulter analyzer, Beckman Coulter) levels were measured from blood samples collected at a single clinic visit. Whole blood was used to extract DNA using Puregene reagents (Gentra Systems). Genetic studies were conducted as described elsewhere^49^, and rs334 genotypes were extracted from exome sequencing datasets as described in Peloso et al.^50^.

### Nairobi, Kenya

Children aged 10–16 years were recruited from the Nairobi Urban Health and Demographic Surveillance System^51^ as part of studies investigating the relationship between sickle cell trait, malaria and blood pressure^52,53^. Nairobi is located at a high altitude (1,800 m above sea level), and there is no evidence of malaria transmission there^54^. Population-wide censuses are conducted four times a year within the study area^51^. Using census data, we selected all school children aged 10–16 years who had a continuous record of residence since birth and had therefore had minimal exposure to malaria. To increase our efficiency in recruiting participants with sickle cell trait, we limited our recruitment to those who identified themselves as genetically descended from ethnic groups whose ancestral residence was in regions endemic for malaria (for example, Luhya, Luo, Teso, Mijikenda). The frequency of sickle cell trait is much higher in these ethnic groups^55^. We measured ferritin, CRP (ILab systems immunoturbidimetric assay, Instrumentation Laboratory) and hemoglobin (Coulter analyzer, Beckman Coulter) levels from stored blood samples. Sickle cell trait was typed by PCR^35,36^ using DNA extracted with the Qiagen DNA Blood Mini kit (Qiagen).

## Study of hospitalized children

### Kilifi hospital-based study

We measured hepcidin levels in children with severe malaria admitted to Kilifi County Hospital^56^. Sixty-two samples were randomly selected. Hepcidin levels were measured using the Hepcidin-25 (human) EIA Bachem kit and harmonized to DRG hepcidin values^43^. Severe malaria was diagnosed as *P. falciparum* parasites in the blood film plus clinical features of severe malaria, including hemoglobin levels <50gl^-1^, a hematocrit level of <15% (for severe malarial anemia) or a Blantyre coma score of 3 (for cerebral malaria).

## Genotyping of sickle cell trait in the VaccGene study

Sickle cell trait (rs334) single-nucleotide polymorphisms (SNPs) were directly genotyped in the VaccGene populations (in Uganda, Burkina Faso and South Africa) using the HumanOmni 2.5M-8 (‘octo’) BeadChip array version 1.1 (Illumina) (*n =648*) and the Illumina Multi-Ethnic Global Array (*n =197*) performed by the genotyping core facilities at the Wellcome Trust Sanger Institute. Genomic DNA underwent whole-genome amplification and fragmentation before hybridization to locus-specific oligonucleotides bound to silica beads with a 3-μm diameter. Fragments were extended by single-base extension to interrogate the variant by incorporating a labeled nucleotide, enabling a two-color detection (Illumina, 2013, https://emea.illumina.com/content/dam/illumina-marketing/documents/products/brochures/datasheet_omni_whole-genome_arrays.pdf). Genotypes were called from intensities using two clustering algorithms (Illuminus and GenCall) in GenomeStudio version 2.0.5 (Illumina) incorporating data from proprietary predetermined genotypes. Details of genotyping and quality control are described elsewhere^46^. The variant rs334 was retained in all datasets following stringent quality control processes^46^.

## Definitions

ID was defined according to WHO recommendation as ferritin levels <12µgl^-1^ or <30µgl^-1^ in the presence of inflammation (defined as CRP>5 mgl^-1^, ACT>0.6gl^-1^ or AGP>1 gl^-1^) in children <5 years or<15µgl^-1^ in children ≦5 years^15^. Anemia was defined as hemoglobin levels<110gl^-1^ in children aged<5 years or hemoglobin levels<115gl^-1^ in children ≦5 years^17^. In the JHS and Nairobi,ID was defined as ferritin levels<30µgl^-1^. In the JHS, anemia was defined as hemoglobin levels<120gl^-1^ in women or<130gl^-1^ in men^16,17^. IDA was defined as the presence of ID and anemia^17^. Malaria parasitemia was defined as a blood slide positive for asexual *P. falciparum* parasites. Underweight was defined as weight-for-age *z* score< -2 using WHO Growth Standards^57^.

## Regression correction

As ferritin is an acute-phase reactant and correlates positively with inflammatory markers^58,59^, we further defined ID after regression correction for the effect of inflammation on ferritin levels as proposed by the Biomarkers Reflecting Inflammation and Nutritional Determinants of Anemia project^19,60^. This approach predicts what the ferritin level would have been in the absence of inflammation or infection and then applies the corrected values to estimate the prevalence of ID. The regression-correction approach followed a three-step process. In the first step, internal reference values for inflammatory markers (CRP or ACT) were defined as the tenth percentile. CRP levels were measured in all studies except in The Gambia, where ACT levels were measured. In addition to CRP, AGP was measured in Malawi, Ghana, DRC and Cameroon; however, because CRP was positively correlated with AGP, and also for consistency, we only corrected for CRP in these studies. To avoid overcorrection for very low levels of inflammatory markers, only participants with CRP or ACT values above the tenth percentile had their ferritin values subtracted from observed values in equation (1)below^19^. In the second step, univariable linear regression models were applied to each study, with ferritin as the dependent variable, to estimate regression coefficients for the crude association between inflammatory marker and ferritin (*β*).In the third step, the regression coefficients estimated in step 2 were used to calculate adjusted ferritin values using equation (1). Ferritin and inflammatory markers were applied in the equations after ln transformation.

(1) $${\text{Ferritin}}_{\text{adjusted}}{\;=\;\text{Ferritin}}_{\text{unadjusted}}-\;\beta \left({\text{CRP}\;\text{or}\;\text{CT}}_{\text{obs}}-{\text{CRP}\;\text{or}\;\text{ACT}}_{\text{ref}}\right)$$

‘Obs’ is the observed value, and ‘ref’ is the reference value.

We then defined ID using the regression-corrected unlogged ferritin value (that is, adjusted for the effects of inflammation) using the same thresholds that were applied to the uncorrected ferritin levels in the WHO recommendations (that is, ferritin levels <12µgl^-1^ in children <5 years or <15µgl^-1^ in children aged ≥5 years^15)^.

## Systematic review and meta-analysis of common genetic polymorphisms associated with malaria risk

Mendelian randomization is an instrumental variable analysis that reduces biases from confounding and reverse causation by using genetic variants to proxy the exposure (for example, malaria) and estimate a causal effect of that exposure on the outcome (for example, ID). To perform Mendelian randomization analyses, valid instrumental variables are required. The instrumental variables must be associated with the exposure of interest, and the effect size can be obtained from association studies. We therefore performed a systematic review and meta-analysis of published studies in African populations to determine the overall effect of common genetic polymorphisms that are associated with uncomplicated malaria. These polymorphisms included those influencing sickle cell trait,α-thalassemia and G6PD. We performed the search in the PubMed database for papers published before 20 March 2020. For sickle cell trait, the search terms included (‘malaria’ (title/abstract) or ‘malaria/blood’ (MAJR) or ‘malaria/ genetics’ (Medical Subject Headings (MeSH) terms) or ‘Malaria’ (MeSH) or ‘Malaria, Falciparum’ (MeSH) or ‘Plasmodium falciparum’ (MeSH)) and (‘HbAS’ (title/abstract) or ‘sickle cell trait’ (title/abstract) or ‘sickle cell trait/genetics’ (MeSH terms) or ‘sickle cell trait/blood’ (MeSH terms) or ‘Hemoglobin, Sickle’ (MeSH) or ‘Sickle Cell Trait’ (MeSH) and ‘Africa’ (MeSH terms)), yielding 594 articles. For α-thalassemia, the search terms included (‘malaria’ (title/abstract) or ‘malaria/ blood’ (MAJR) or ‘malaria/genetics’ (MeSH terms) or ‘Malaria’ (MeSH) or ‘Malaria, Falciparum’ (MeSH) or ‘Plasmodium falciparum’ (MeSH)) and (‘alpha-thalassemia’ (title/abstract) or ‘alpha-thalassemia/genetics’ (MeSH terms) or ‘alpha-thalassemia/ blood’ (MeSH terms) or ‘α-thalassemia’ (MeSH)) and Africa’ (MeSH terms) and yielded 65 articles. The search terms for G6PD included (‘malaria’ (title/abstract) or ‘malaria/blood’ (MAJR) or ‘malaria/genetics’ (MeSH terms) or ‘Malaria’ (MeSH) or ‘Malaria, Falciparum’ (MeSH) or ‘Plasmodium falciparum’ (MeSH)) and (‘G6PD’ (title/abstract) or ‘glucose-6-phosphate-dehydrogenase’ (title/abstract) or ‘glucose-6-phosphate-dehydrogenase/genetics’ (MeSH terms) or ‘glucose-6-phosphate-dehydrogenase/blood’ (MeSH terms)) and ‘Africa’ (MeSH terms) and yielded 187 articles.

We restricted our analysis to studies conducted in Africa, as our outcome of interest (ID) was measured in African populations. We also focused on studies reporting IRR, as children are repeatedly infected with malaria; and, therefore, an IRR provides a better estimate of the true malaria risk reduction attributable to genetic polymorphisms. Studies included in the meta-analysis are shown in Supplementary Table 2.

## Statistical analysis

All statistical analyses were conducted using Stata 13.0 (StataCorp). All measurements were taken from distinct samples. We conducted both cohort-specific and pooled analyses. When appropriate, we computed percentages, geometric means and deciles. We used two-tailed Student’s *t*-tests to test for differences in means of log_*e*_-transformed hepcidin concentrations between children with and without malaria parasitemia or between those with severe malaria and those without parasitemia. When appropriate, we fitted adjusted logistic regression models to determine the effect of sickle cell trait on ID. The cohort-specific ORs were meta-analyzed assuming fixed effects because there was little evidence of heterogeneity between studies. We used meta-analysis to determine the overall effect size and also to identify potential heterogeneity that may have been introduced by different populations and laboratory facilities. *A P* value <0.05 was considered statistically significant. All *P* values reflect two-tailed tests. Individuals with sickle cell disease (HbSS) were not included in the analyses, as numbers were few and there is little evidence suggesting that HbSS protects against uncomplicated malaria.

We used the online (http://cnsgenomics.com/shiny/mRnd/) Mendelian randomization power calculator to calculate sample size. The sample size of 7,453 had power above 80% given the observed OR of the outcome variable per s.d. of the exposure variable of 2.65, 2% variation of clinical malaria that is explained by sickle cell trait^61,62^, 27% prevalence of ID and a type I error rate of 0.05.

**Fig.1:**
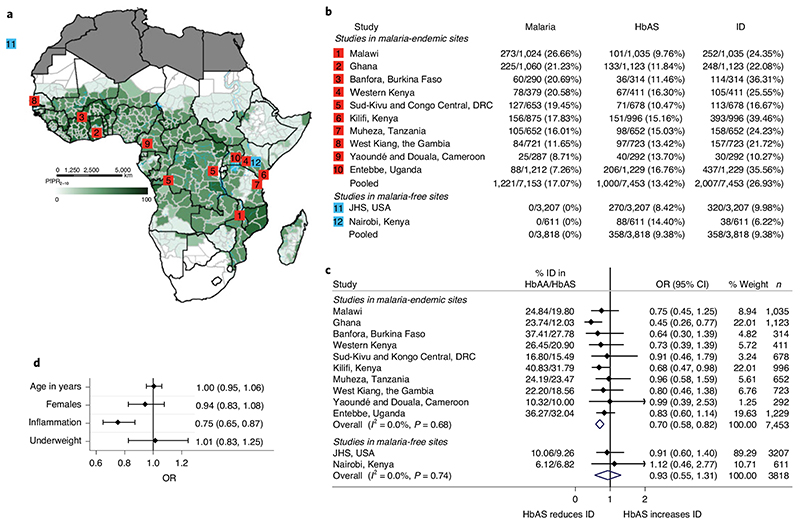
Sickle cell trait (HbAS) is associated with protection from ID. **a**, Map of malaria in Africa showing the predicted posterior predictions of age-standardized *P. falciparum* prevalence (PfPR_2-10_) taken from Snow et al.^3^ and the location of the current study sites with data on sickle cell trait (HbAS). The map was reproduced with permission.**b**, Prevalence (%) of malaria parasitemia, HbAS and ID for each study site.**c**, Summary results of the effect of HbAS on ID by study site. ‘Overall’ represents a fixed-effect meta-analysis of study-specific odds ratios (ORs).**d**, ORs for the associations between sickle cell trait and the variables age, sex, inflammation and underweight. For age, sex and inflammation, *n* =7,453 biologically independent samples were used, and for underweight, *n* = 6,428 biologically independent samples were used. Inflammation was defined as CRP >5 mgl^-1^ or ACT > O.6gl^-1^ or AGP >lgl^-1^. Underweight was defined as a WHO 2006 reference weight-for-age *z* score <-2. All error bars indicate 95% CIs. HbAA, normal hemoglobin.

To investigate whether uncomplicated malaria is causally associated with ID, a two-sample Mendelian randomization^63^ was conducted using the ‘mrrobust’ software package^64^ in Stata 13.0. This is a Wald ratio involving two estimates: the SNP –outcome effect divided by the SNP –exposure effect, in this case, the HbAS (rs334) –ID effect divided by the HbAS (rs334) –malaria effect. The first estimate (SNP –outcome) for sickle cell trait (HbAS, rs334) on ID was from the above-described community-based cohorts in which the overall log odds estimate was determined using the meta-analyzed cohort-specific estimates (Fig. 1c). The second estimate (SNP–exposure) was from the meta-analyzed overall log IRR of sickle cell trait on uncomplicated malaria (Extended Data Fig. 4). A causal log odds estimate equation (2), which is the ratio of the first estimate and the second estimate, was computed^63^. The causal OR was obtained by exponentiating the causal log odds estimate and interpreted as change in ID per unit increase in the log incidence rate of malaria. We further calculated the effect on ID of reducing malaria incidence by half. This was obtained by multiplying the causal log odds by the natural logarithm of 0.5 and then exponentiating the result^20^.

(2) $$\text{Causal}\;\text{log}\;\text{odds}=\frac{\text{log}\;\text{odds}\;\text{of}\;\text{iron}\;\text{deficiency}\;\text{in}\;\text{HbAS}}{\text{log}\;\text{incidence}\;\text{rate}\;\text{ratio}\;\text{of}\;\text{malaria}\;\text{in}\;\text{HbAS}}$$

To determine whether sickle cell trait influences ID independently of malaria, we conducted sensitivity or zero relevance point analyses in negative controls, that is, populations that were not exposed to malaria^14^. To do this, we repeated our analyses in two separate populations that were not exposed to malaria including (1) an African American adult population (*n*=3,207) and (2) life-long adolescent residents of Nairobi (*n*=611), where there is no evidence of malaria transmission^54^. As this analysis suggested that an effect was absent in populations that were not exposed to malaria, this allowed us to interpret the effect among children exposed to malaria.

## Ethical approvals

Individual study site ethical approvals were obtained for the Kilifi, Kenya study (by the Scientific Ethics Review Unit of the Kenya Medical Research Institute (KEMRI/SERU/CGMR-C/046/3257/2983)), the Entebbe, Uganda study (locally by the Uganda Virus Research Institute (GC/127/12/07/32) and the Uganda National Council for Science and Technology (MV625) and in the UK by the London School of Hygiene and Tropical Medicine (A340) and the Oxford Tropical Research (OTR) (39-12, 42-14 and 37-15) Ethics Committees), the Banfora, Burkina Faso study (by Ministere de la Recherche Scientifique et de l’Innovation in Burkina Faso (2014-12-151) and the OTR Ethics Committees(4112)), the Soweto, South Africa study (by the University of Witwatersrand Human Research (M130714) and the OTR Ethics Committees (1042-13 and 42-14)) and the West Kiang, The Gambia study (by the Gambian Government, Medical Research Council Ethics Committee (874/830)). For the additional seven study sites, individual study data transfer agreements were signed with the responsible study and/or institution’s principal investigator and the KEMRI-Wellcome Trust Research Programme study principal investigator, S.H.A. These studies had ethical approval to share de-identified data for further secondary analyses presented in this study. Informed written consent was obtained from all children’s parents or guardians.

## Reporting Summary

Further information on research design is available in the Nature Research Reporting Summary linked to this article

## Extended Data

**Extended Data Fig.1:**
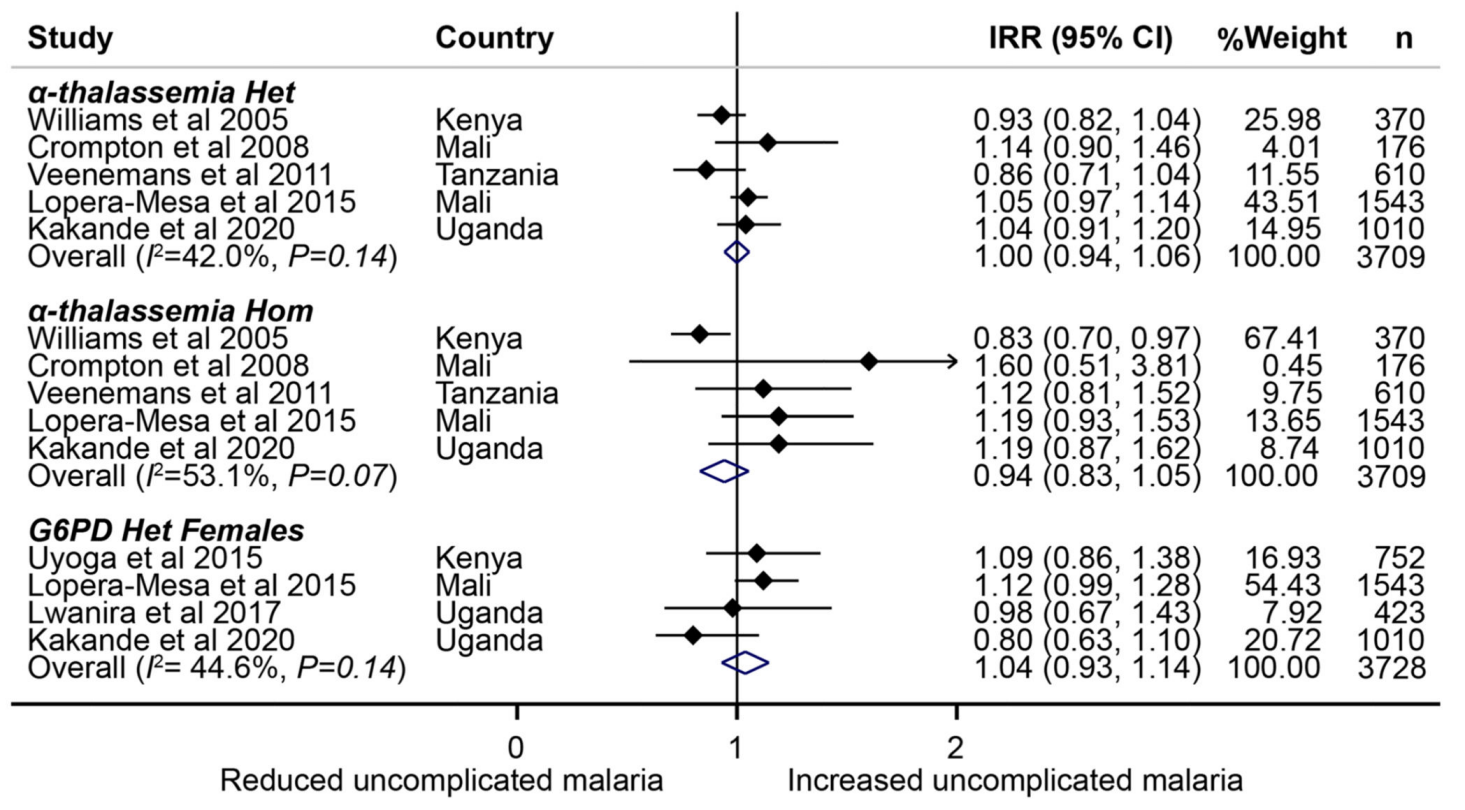
A meta-analysis of previous African studies investigating the effect of α-thalassemia and G6PD A and A-polymorphisms on uncomplicated febrile malaria. Overall represents a fixed-effect meta-analysis of study-specific incidence rate ratio (IRR) by genetic polymorphism. Error bars indicate 95% confidence intervals. n shows the number of individuals included in the analysis. Few studies included G6PD homozygous females and numbers were small (Table S2). Het, heterozygous; Hom, homozygous.

**Extended Data Fig.2:**
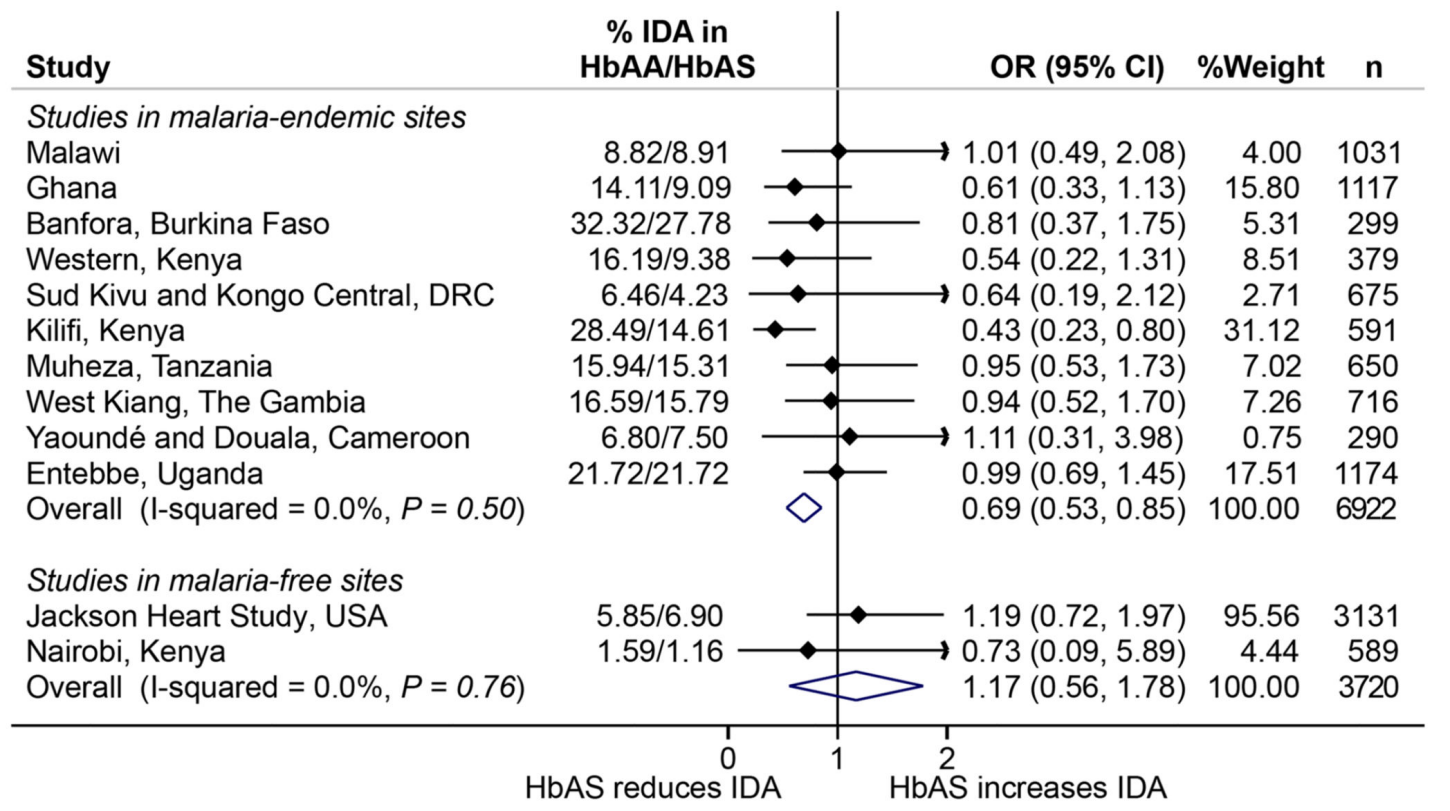
A meta-analysis of the effect of sickle cell trait on iron deficiency anemia (IDA). Overall represents a fixed-effect meta-analysis of cohort-specific odds ratios. Error bars indicate 95 % confidence intervals. n shows the number of individuals included in the analysis. Numbers for IDA are fewer compared to those for ID since not all children had hemoglobin concentrations measured.

**Extended Data Fig.3:**
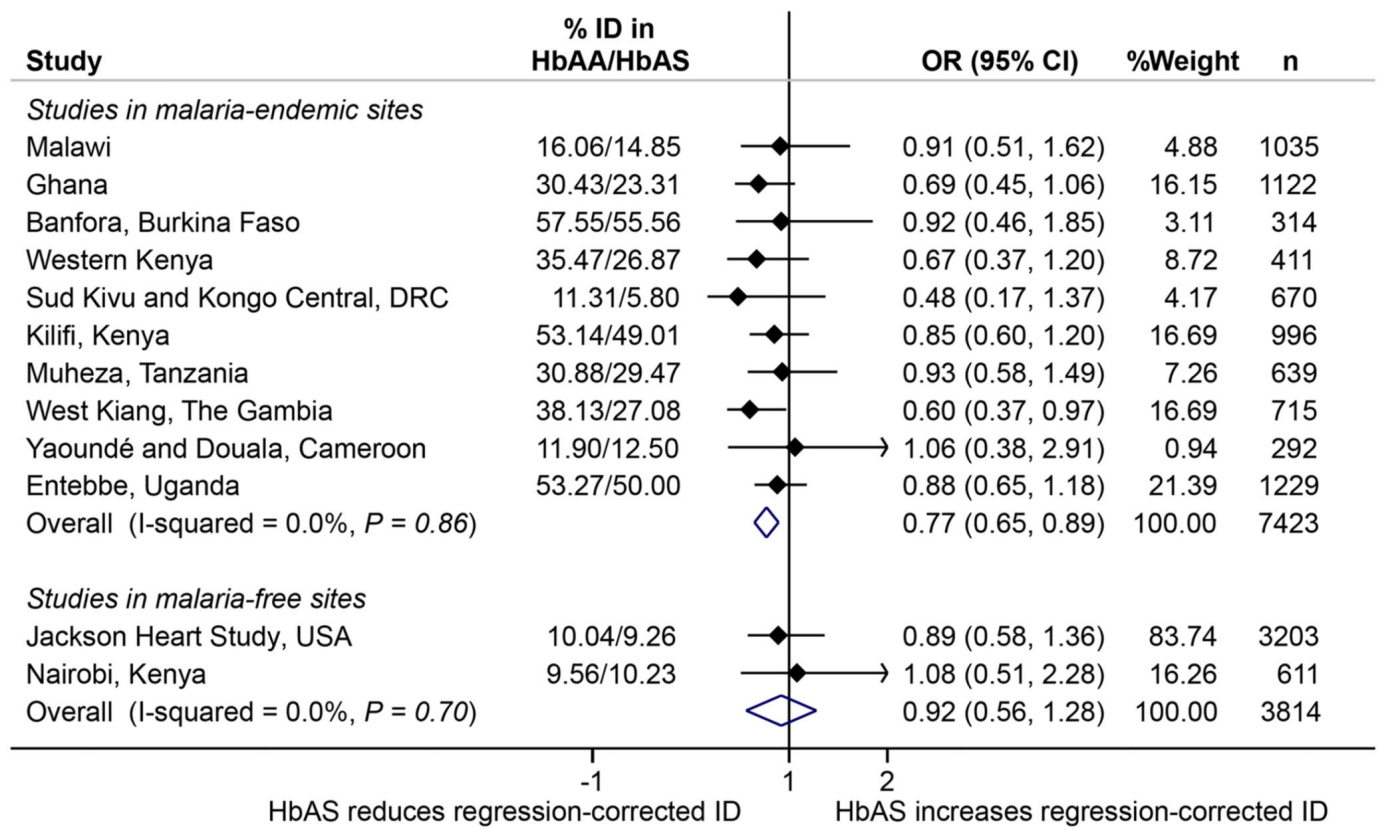
A meta-analysis of the effect of sickle cell trait on iron deficiency (ID) regression-corrected for inflammation. ID was defined using ferritin levels adjusted for the effects of inflammation using a regression-correction approach as developed by BRINDA. Overall represents a fixed-effect meta-analysis of cohort-specific odds ratios. Error bars indicate 95 % confidence intervals. n shows the number of individuals included in the analysis.

**Extended Data Fig.4:**
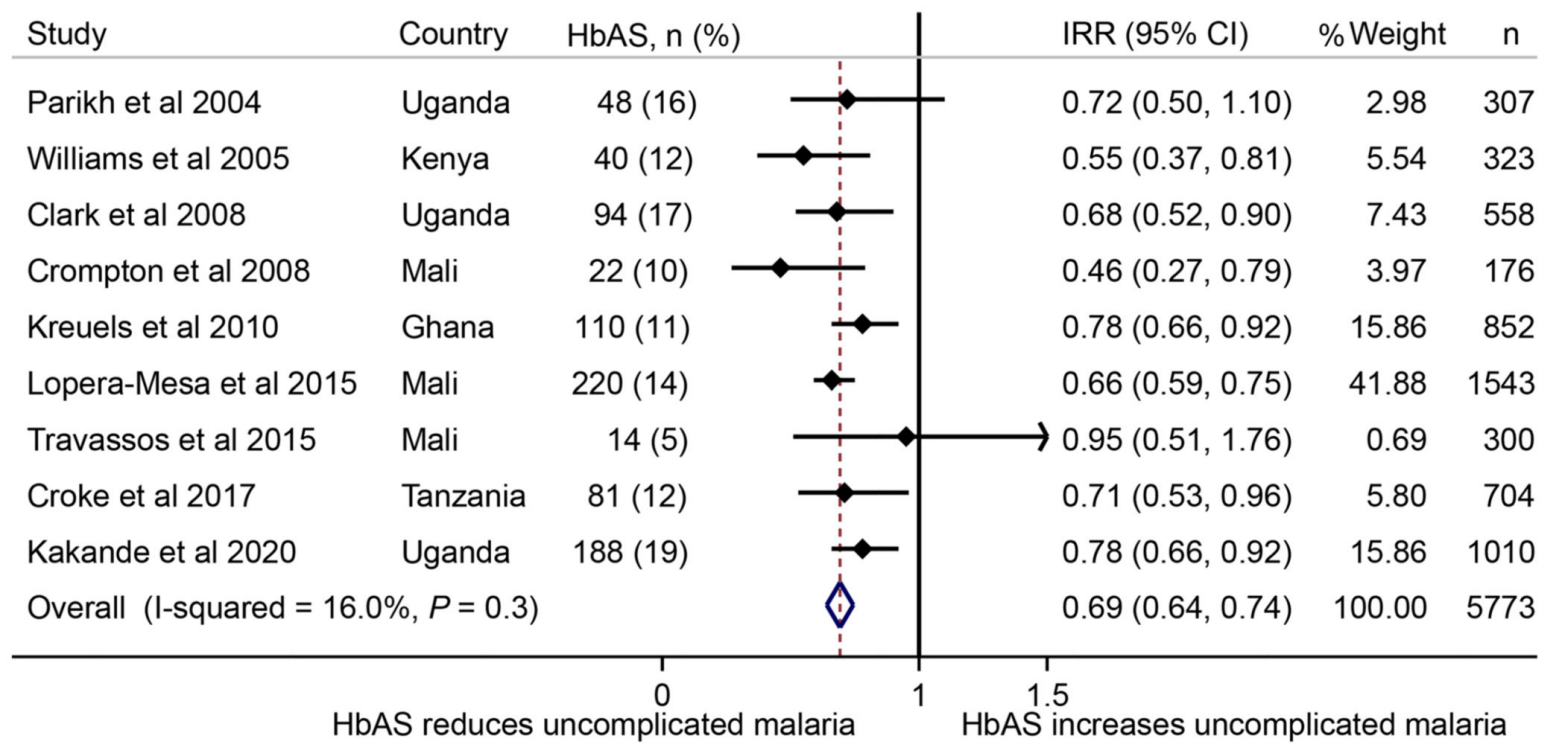
A meta-analysis of the effect of sickle cell trait on uncomplicated febrile malaria. Overall represents a fixed-effect meta-analysis of study-specific incidence rate ratio (IRR). Error bars indicate 95 % confidence intervals. n shows the number of individuals included in the analysis.

**Extended Data Fig.5:**
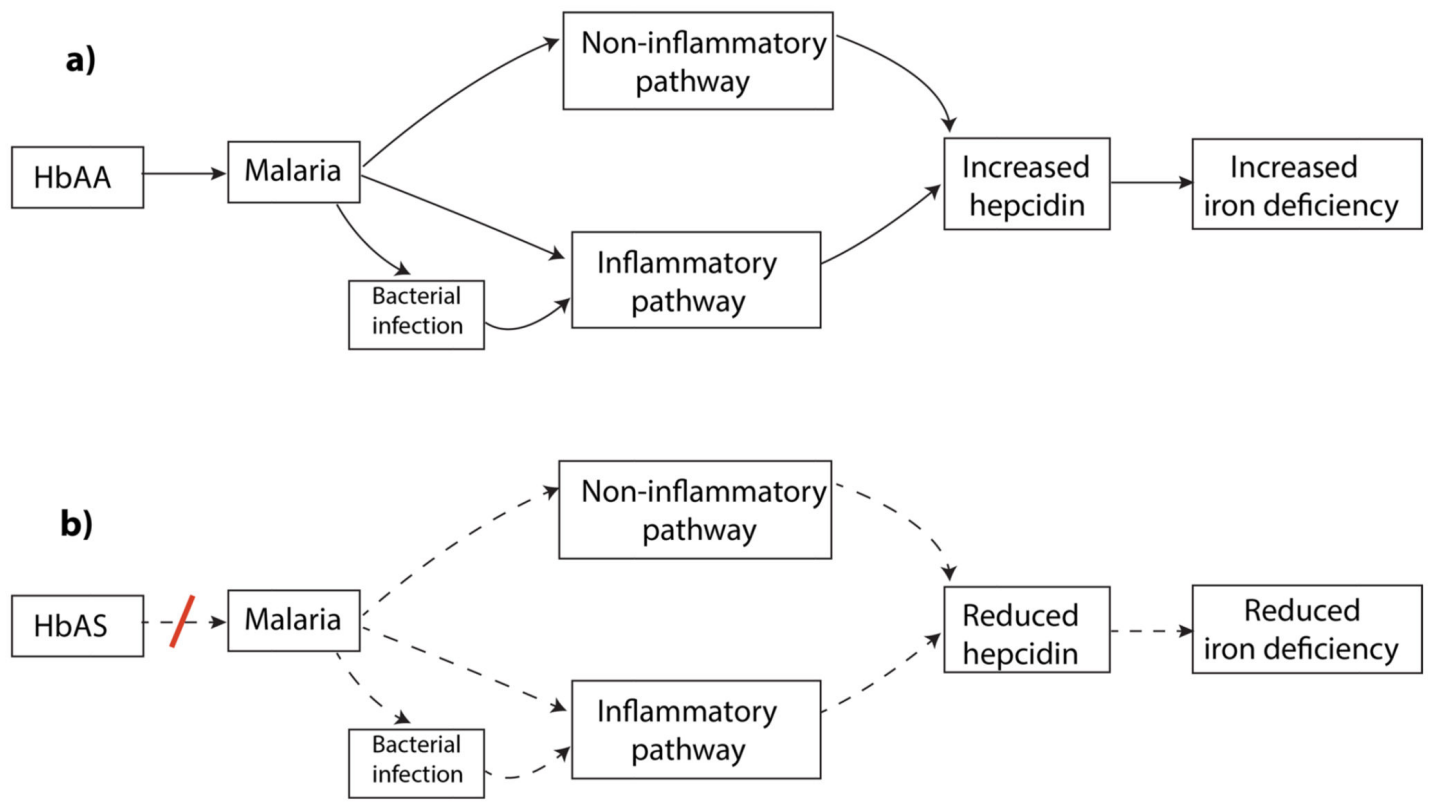
How HbAS,a genetic proxy for malaria exposure,may protect children from iron deficiency. **a**, Individuals carrying normal beta hemoglobin gene (HbAA) are not protected from malaria. Malaria up-regulates production of hepcidin through inflammatory and non-inflammatory pathways and by increasing the prevalence of other infections. Hepcidin in turn blocks iron absorption.**b**,Sickle cell trait (HbAS) partially protects individuals from malaria infection, therefore inflammation is reduced leading to reduced hepcidin stimulation and increased iron absorption.

**Extended Data Fig.6:**
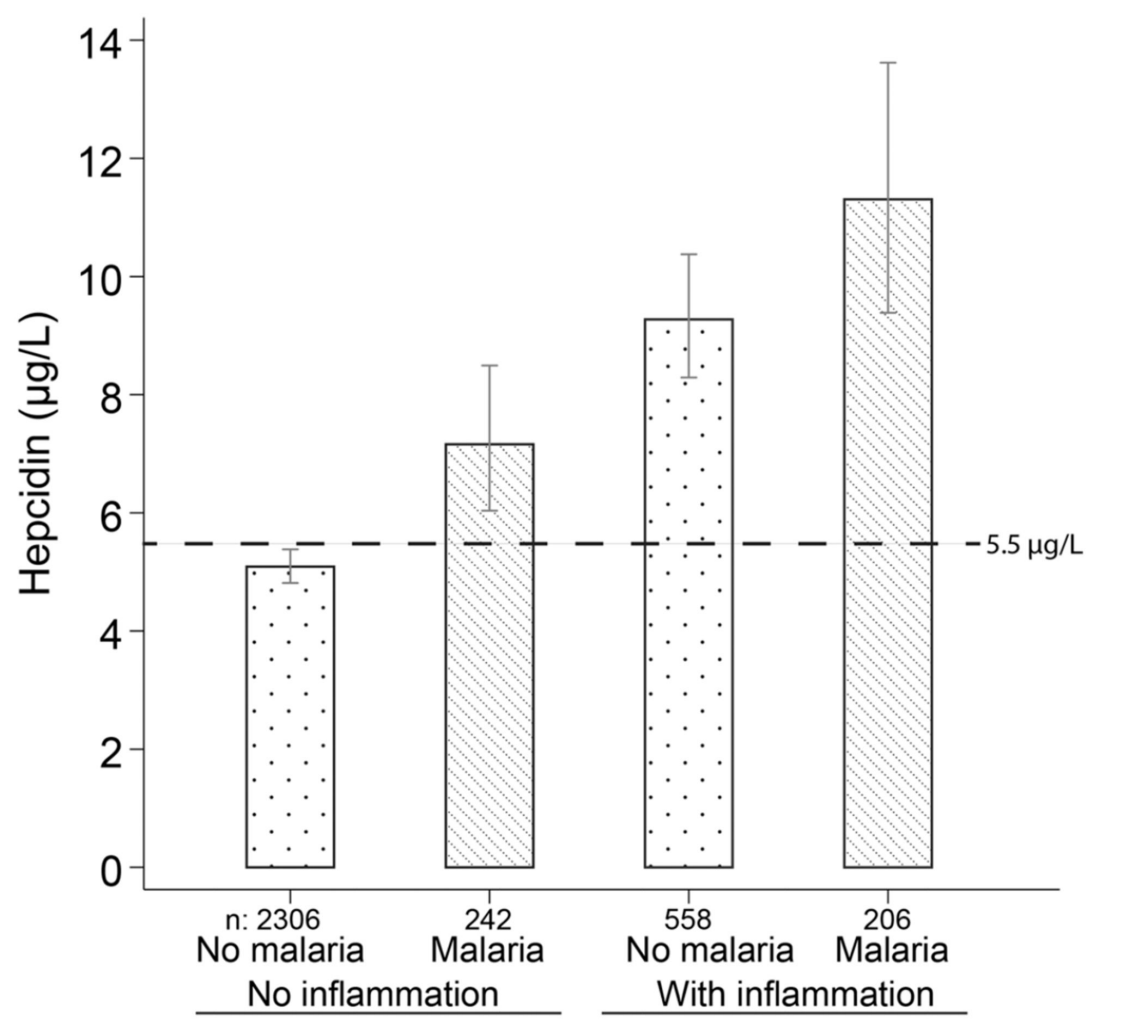
Relationship between geometric mean hepcidin concentrations, malaria parasitemia and inflammation. Error bars indicate 95 % confidence intervals. n indicates biologically independent samples. Horizontal dotted line indicates the threshold of hepcidin above which iron absorption is inhibited (5.5 μg/L). Inflammation was defined as CRP >5 mg/L, ACT >O.6g/L or AGP >1 g/L. Malaria was defined as a blood slide positive for asexual *P. falciparum* parasites. Hepcidin was measured in the Burkina Faso, Western Kenya, Uganda, The Gambia, and Kilifi, Kenya cohorts.

## Supplementary Material

Supplementary information

## Figures and Tables

**Fig.2:**
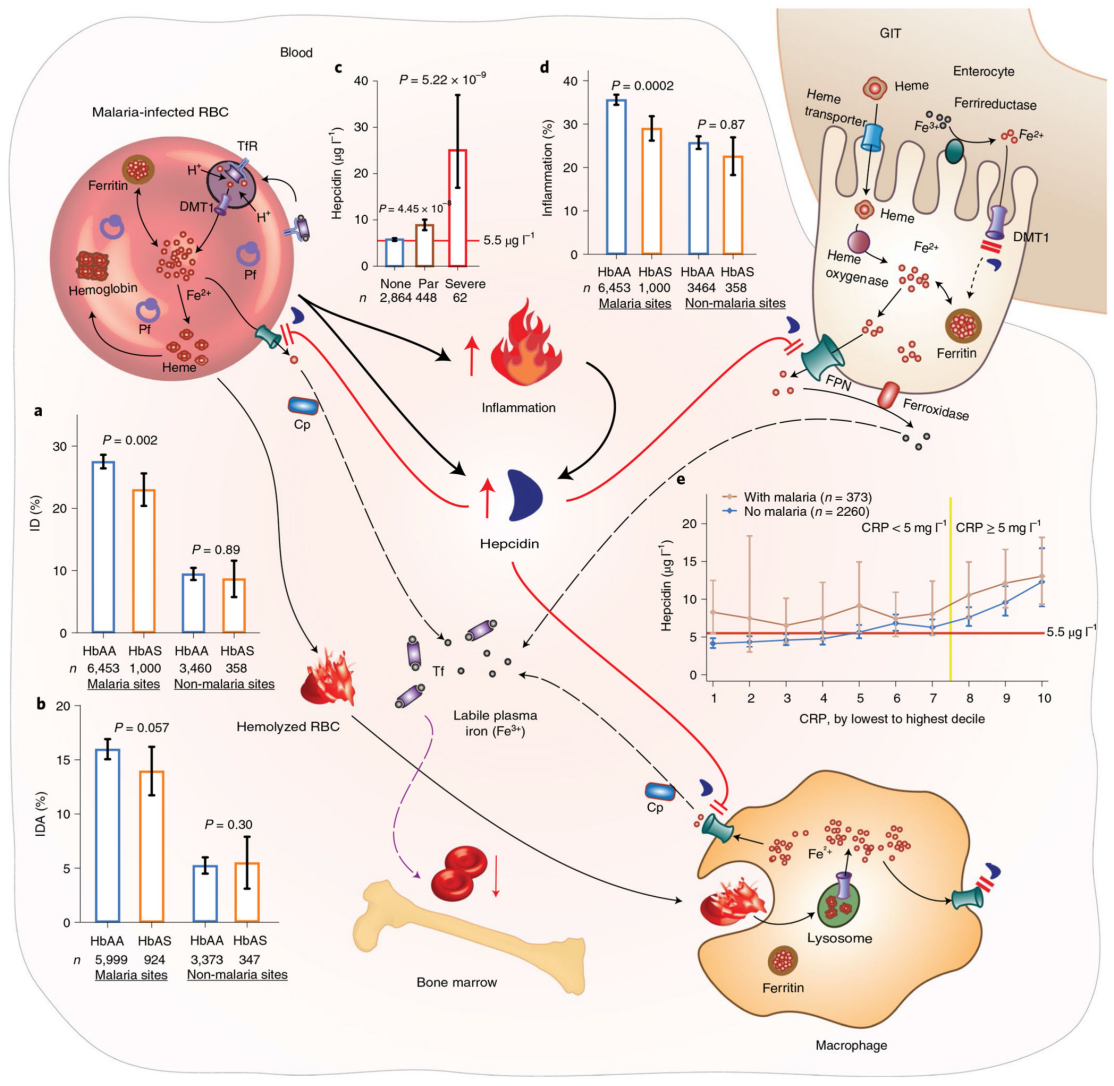
How malaria might cause a hepcidin-mediated blockade of iron absorption and recycling leading to ID. The prevalences (%) of ID (**a**) and IDA (**b**) were lower in African children carrying sickle cell trait (HbAS), a genetic variant that protects against malaria, than in those with normal hemoglobin (HbAA) in malaria-endemic sites but not in sites without malaria. *P* values were derived from logistic regression analyses adjusted for age,sex, inflammation and study site. **c**, Geometric means of hepcidin concentrations were higher in children with malaria parasitemia or severe malaria compared to those without. *P* values were derived from two-tailed Student’s *t*-tests. **d**, Prevalence (%) of inflammation was lower in individuals with HbAS than in those with HbAA in malaria-endemic sites but not in sites without malaria.*P* values were derived from logistic regression analyses adjusted for age, sex and study site. **e**, Geometric means of hepcidin concentrations increase with increasing CRP concentrations and remain above threshold for iron absorption at almost all deciles of CRP in children with malaria parasitemia. The brown line shows children with malaria parasitemia, and the blue line shows those without parasitemia. The yellow line shows the point at which inflammation is clinically diagnosed. The red horizontal line indicates the threshold of hepcidin above which iron absorption is inhibited (5.5μgl^-1^). Data were pooled for malaria-endemic sites and for sites without malaria (Jackson Heart Study (JHS) and Nairobi). Hepcidin levels were measured in the Burkina Faso, Western Kenya, Uganda, The Gambia and Kilifi, Kenya cohorts. CRP levels were measured in all sites except The Gambia.*n* indicates biologically independent samples. All error bars indicate 95% CIs. Cp, ceruloplasmin; DMT1, divalent metal transporter 1; FPN, ferroportin; GIT, gastrointestinal tract; Par, malaria parasitemia; Pf,*P. falciparum;* RBC, red blood cell; Tf, transferrin; TfR, transferrin receptor.

**Table 1:** Characteristics of study participants by site

	Total	Age (years) mean (s.d.)	Female sex, *n* (%)	Inflammation’, *n* (%)	Underweight^b^, *n* (%)
Studies in malaria-endemic sites					
Malawi	1,035	2.75(1.24)	512(49.47)	604(58.36)	134(13.00)
Ghana	1,123	2.67(1.28)	560(49.96)	230(20.48)	177(16.06)
Banfora, Burkina Faso	314	1.86(0.46)	153(48.73)	106(33.76)	57(19.32)
Western Kenya	411	1.85(0.15)	210(51.09)	92(22.38)	32(7.82)
Sud Kivu and Kongo Central, DRC	678	2.41(1.14)	338(49.85)	462(68.14)	141(22.49)
Kilifi, Kenya	996	2.20(1.47)	489(49.10)	261(26.20)	72(26.67)
Muheza, Tanzania	652	1.64 (0.79)	313 (48.01)	370 (56.75)	49 (8.02)
West Kiang, The Gambia	723	3.91 (1.16)	333 (46.06)	107 (14.80)	145 (25.53)
Yaoundé and Douala, Cameroon	292	2.76 (1.05)	143 (49.48)	62 (21.23)	15 (5.21)
Entebbe, Uganda	1,229	2.30 (0.82)	603 (49.06)	291 (23.68)	100 (8.16)
Studies in malaria-free sites					
JHS	3,207	55.57 (12.83)	2,000 (62.36)	940 (29.31)	12 (0.38)
Nairobi, Kenya	611	12.67 (1.17)	277 (45.34)	32 (5.24)	NA
Soweto, South Africa	845	1.01 (0.10)	431 (51.01)	145 (17.16)	NA
Hospitalized children with malaria					
Kilifi hospital-based study	62	1.86 (1.06)	28 (45.16)	62 (100.00)	26 (41.94)

DRC, Democratic Republic of Congo; RCT, randomized controlled trial; NA, not available. inflammation was defined as CRP>5mgl^-1^ or ACT >O.6gl^-1^ or AGP>1gl^-1^. CRP levels were measured in all sites except West Kiang, The Gambia, where only ACT was measured. In addition to CRP, AGP was measured in Malawi, Ghana, DRC and Cameroon. ^b^Underweight was defined as a WHO 2006 reference weight-for-age *z* score <-2. Causes of underweight in African American adults are likely to be different from those in African children.

**Table 2:** Estimates used in Mendelian randomization analyses

Relation	Estimate (s.e.)	*P*	Source
HbAS-ID	-0.36 (0.09)	7.41 ×10^-31^	Meta-analyzed African studies (Fig. 1c)
HbAS-uncomplicated malaria	-0.37 (0.04)	1.33×10^-151^	Meta-analysis of published studies (Extended Data Fig. 4)
MR causal estimate	0.97 (0.24)	0.0001	Calculated as HbAS-ID÷HbAS-malaria

The first estimate, the HbAS-ID estimate, is the natural log odds of the meta-analyzed cohort-specific OR from Fig. 1c. The second estimate, the HbAS-uncomplicated malaria estimate, is the natural log IRR of the meta-analyzed study-specific IRRs from published studies (Extended Data Fig. 4). The Mendelian randomization causal estimate (the causal log odds) is the ratio of the first and second estimates, the Wald ratio. *P* values reflect two-tailed tests. MR, Mendelian randomization; s.e., standard error.

## Data Availability

All data are available in the main text or in the Supplementary Information. Primary individual-level de-identified data for the Kilifi, Kenya; Entebbe, Uganda; Banfora, Burkina Faso; and West Kiang, The Gambia cohorts are available in Harvard Dataverse at https://doi.org/10.7910/DVN/UKGRVJ; applications for access to these data and to the Nairobi dataset can be made through the Data Governance Committee (dgc@kemri-wellcome.org). Data from the 2015–2016 MMS are available from the DHS Program at https://dhsprogram.com/what-we-do/survey/survey-display-483.cfm. The data underlying the results from the Ghana site are owned by the UNICEF Ghana and the Ministry of Health Ghana and contain confidential, identifying information. Data are available from the UNICEF Ghana (accra@unicef.org) for researchers who meet the criteria for access to confidential data. De-identified data from the western Kenya study are available on Open Science Framework at the following link: https://osf.io/dsrv2/. Data from the Sud Kivu and Kongo Central, DRC studies are available at https://doi.org/10.7910/DVN/RNWYR8. All data used in the analysis of the MOMS Project cohort (Muheza, Tanzania) are available under human data transfer agreement for purposes of reproducing or extending the analysis. Data for the Cameroon study are available upon reasonable request to the survey representative A. Ndjebayi (andjebayi@hki.org), Helen Keller International, Cameroon Office, Rue 1771, Bastos, BP 14227, Yaoundé. All JHS data are available at https://www.jacksonheartstudy.org/Research/Study-Data/Data-Access. Additionally, much of the JHS phenotype data are available at BioLINCC (https://biolincc.nhlbi.nih.gov/studies/jhs/), and data for genetic analyses are available through dbGaP at phs000286. Source data are provided with this paper.
