# Supplementary material for: Malaria is a cause of iron deficiency in African children
Source: Nat Med. Author manuscript; Available in PMC 2021 Apr 23. (PMC7610676; doi:10.1038/s41591-021-01238-4)
Supplement: Supplementary information [file EMS121305-supplement-Supplementary_information.pdf]

nature portfolio

https://doi.org/10.1038/s41591-021-01238-4

Supplementary information

Malaria is a cause of iron deficiency in African children

In the format provided by the authors and unedited

Supplementary Table 1. Published studies on the incidence rate ratio of uncomplicated febrile malaria in children with sickle cell trait, α-thalassemia, and G6PD polymorphisms in Africa

Study	Country	Sample size	Age	IRR (95% CI)
Sickle cell trait				
Parikh *et al* 2004[1]	Uganda	307	6 months - 5 years	0.72 (0.50, 1.10)
Williams *et al* 2005[2]	Kenya	323	<8 years	0.55 (0.37, 0.81)
Clark *et al* 2008[3]	Uganda	558	1 - 10 years	0.68 (0.52, 0.90)
Crompton *et al* 2008[4]	Mali	176	2 - 10 years	0.46 (0.27, 0.79)
Kreuels *et al* 2010[5]	Ghana	852	3 months - 2 years	0.78 (0.66, 0.92)
Lopera-Mesa *et al* 2015[6]	Mali	1543	6 months - 17 years	0.66 (0.59, 0.75)
Lwanira *et al* 2015[7]*	Uganda	413	3 - 9 years	0.78 (0.76, 1.43)
Travassos *et al* 2015[8]	Mali	300	0 - 6 years	0.95 (0.51, 1.76)
Croke *et al* 2017[9]	Tanzania	767	0 - 19 years	0.71 (0.53, 0.96)
Kakande *et al* 2020[10]	Uganda	1010	6 - 10 years	0.78 (0.66, 0.92)
α-thalassemia het				
Williams *et al* 2005[11]	Kenya	370	<11 years	0.93 (0.82, 1.04)
Crompton *et al* 2008[4]	Mali	176	2-10 years	1.14 (0.90, 1.46)
Veenemans *et al* 2011[12]	Tanzania	610	6 months - 5 years	0.86 (0.71, 1.04)
Lopera-Mesa *et al* 2015[6]	Mali	1543	6 months - 17 years	1.05 (0.97, 1.14)
Kakande *et al* 2020[10]	Uganda	1010	6 - 10 years	1.04 (0.91, 1.20)
α-thalassemia hom				
Williams *et al* 2005[11]	Kenya	370	<11 years	0.83 (0.70, 0.97)
Crompton *et al* 2008[4]	Mali	176	2-10 years	1.60 (0.51, 3.81)
Veenemans *et al* 2011[12]	Tanzania	610	6 months - 5 years	1.12 (0.81, 1.52)
Lopera-Mesa *et al* 2015[6]	Mali	1543	6 months - 17 years	1.19 (0.93, 1.53)
Kakande *et al* 2020[10]	Uganda	1010	6 - 10 years	1.19 (0.87, 1.62)
G6PD het females				
Uyoga *et al* 2015[13]	Kenya	752	<10 years	1.09 (0.86, 1.38)
Lopera-Mesa *et al* 2015[6]	Mali	1543	6 months - 17 years	1.12 (0.99, 1.28)
Lwanira *et al* 2017[14]	Uganda	423	<9 years	0.98 (0.67, 1.43)
Kakande *et al* 2020[10]	Uganda	1010	6 - 10 years	0.80 (0.63, 1.10)
G6PD hom females[†]				
Lopera-Mesa *et al* 2015[6]	Mali	1543	6 months - 17 years	0.51 (0.29, 0.90)
Lwanira *et al* 2017[14]	Uganda	423	<9 years	1.38 (0.31, 6.03)
Kakande *et al* 2020[10]	Uganda	1010	6 - 10 years	0.89 (0.51, 1.57)

*This study was excluded from meta-analysis because the confidence intervals appeared unreliable.
[†]Studies included only few G6PD homozygous females (n = 13 in Lopera-Mesa *et al* 2015, 4 in Lwanira *et al* 2017, and 12 in Kakande *et al* 2020).
Het, heterozygous; hom, homozygous

Supplementary Table 2. Effect of sickle cell trait (HbAS) on iron deficiency before and after adjusting for α-thalassemia

Study	HbAS		HbAS adjusted for α-thalassemia	
	**n**	**OR (95% CI)**	**n**	**OR (95% CI)**
Malawi	1035	0.75 (0.45, 1.25)	1030	0.76 (0.45, 1.26)
Ghana	1123	0.45 (0.26, 0.77)	1045	0.46 (0.26, 0.80)
Western, Kenya	411	0.73 (0.39, 1.39)	409	0.73 (0.38, 1.38)
Sud Kivu and Kongo Central, DRC	678	0.91 (0.46, 1.79)	381	0.66 (0.22, 1.98)
Kilifi, Kenya	996	0.68 (0.47, 0.98)	991	0.68 (0.47, 0.98)
Muheza, Tanzania	652	0.96 (0.58, 1.59)	616	0.99 (0.59, 1.70)
Yaoundé and Douala, Cameroon	292	0.99 (0.39, 2.53)	270	0.99 (0.32, 3.08)
Overall (fixed-effects meta-analysis)	4046	0.76 (0.58, 0.93)	3697	0.74 (0.56, 0.92)

Logistic regression results of the effect of HbAS on ID by study site in overall fixed-effects meta-analysis of children in malaria-endemic areas, and the effect of HbAS on ID after adjusting for α-thalassemia. The interaction term between HbAS and α-thalassemia in predicting iron deficiency was 0.82 (95% CI 0.61, 1.11); Two-tailed $P = 0.20$

References

1. Parikh, S., Dorsey, G. & Rosenthal, P. J. Host polymorphisms and the incidence of malaria in Ugandan children. *Am. J. Trop. Med. Hyg.* **71**, 750–753 (2004).
2. Williams, T. N. *et al.* Sickle cell trait and the risk of Plasmodium falciparum malaria and other childhood diseases. *J. Infect. Dis.* **192**, 178–186 (2005).
3. Clark, T. D. *et al.* Factors determining the heterogeneity of malaria incidence in children in Kampala, Uganda. *J. Infect. Dis.* **198**, 393–400 (2008).
4. Crompton, P. D. *et al.* Sickle cell trait Is associated with a delayed onset of malaria: implications for time-to-event analysis in clinical studies of malaria. *J. Infect. Dis.* **198**, 1265–1275 (2008).
5. Kreuels, B. *et al.* Differing effects of HbS and HbC traits on uncomplicated falciparum malaria, anemia, and child growth. *Blood* **115**, 4551–4558 (2010).
6. Lopera-Mesa, T. M. *et al.* Effect of red blood cell variants on childhood malaria in Mali: A prospective cohort study. *Lancet Haematol.* **2**, e140–e149 (2015).
7. Lwanira, C. N., Mukasa, M. K., Swedberg, G. & Kironde, F. Frequency of RANTES gene polymorphisms and their association with incidence of malaria: A longitudinal study on children in Iganga district, Uganda. *Malar. J.* **14**, 341 (2015).
8. Travassos, M. A. *et al.* Hemoglobin C trait provides protection from clinical falciparum malaria in Malian children. *J. Infect. Dis.* **212**, 1778–1786 (2015).
9. Croke, K. *et al.* Relationships between sickle cell trait, malaria, and educational outcomes in Tanzania. *BMC Infect. Dis.* **17**, 568 (2017).
10. Kakande, E. *et al.* Associations between red blood cell variants and malaria among children and adults from three areas of Uganda: A prospective cohort study. *Malar. J.* **19**, 21 (2020).

11. Williams, T. N. *et al.* Negative epistasis between the malaria-protective effects of α+-thalassemia and the sickle cell trait. *Nat. Genet.* **37**, 1253–1257 (2005).
12. Veenemans, J. *et al.* Effect of α-thalassaemia on episodes of fever due to malaria and other causes: a community- based cohort study in Tanzania. *Malar. J.* **10**, 280 (2011).
13. Uyoga, S. *et al.* Glucose-6-phosphate dehydrogenase deficiency and the risk of malaria and other diseases in children in Kenya: A case-control and a cohort study. *Lancet Haematol.* **2**, e437–e444 (2015).
14. Lwanira, C. N., Kironde, F., Kaddumukasa, M. & Swedberg, G. Prevalence of polymorphisms in glucose-6-phosphate dehydrogenase, sickle haemoglobin and nitric oxide synthase genes and their relationship with incidence of uncomplicated malaria in Iganga, Uganda. *Malar. J.* **16**, 322 (2017).
